# White spaces, music notation and the facilitation of sight-reading

**DOI:** 10.1038/s41598-019-41445-1

**Published:** 2019-03-28

**Authors:** Arild Stenberg, Ian Cross

**Affiliations:** Centre for Music and Science, Faculty of Music, University of Cambridge, West Road, Cambridge, CB3 9DP UK

## Abstract

The use of interword separation has consistently been proven to enhance fluency in reading language scripts. At the same time, neurophysiological evidence has shown that music and language scripts can activate very similar neural circuitry that integratively encodes the symbols that comprise them. By analogy to interword separations in language, we hypothesize that visual separation cues in musical scores should facilitate music reading. We report an experiment in which separating short fragments of musical discourse by vertical white gaps in the notation enhanced sight-reading fluency by significantly reducing the number of mistakes that musicians made when reading the scores without previous preparation. These results are in accordance with a view of music reading as sharing cognitive strategies with language reading; they have significant implications for our understanding of the acquisition of musical literacy and for the design of musical scores, and for our knowledge of the sense-making processes involved in reading in general.

## Introduction

The initial research question motivating this study was whether or not the readability of musical scores could be improved so as to enhance musicians’ capacity to perform fluently when reading with minimal preview, without having to invent and implement a wholly novel notation or system of symbols.

The reading of musical scores has historically been considered something of an expert domain, linked to knowledge of repertoire, idiom, and musical gestures. Studies of motoric responses to the difficulty of musical passages have shown that expertise in music sight-reading (reading a score without preview of its materials) depends on the availability of rule-governed responses, acquired through long years of practice, that are triggered by familiar visual patterns in a score^1^. It should be noted that, from its inception, Western music notation was not designed to be used for performance at first sight. The form of musical notation employed in Western musical practice emerges from a long tradition of use in liturgical contexts that relied heavily on a comprehensive knowledge of local repertoire and performance context, with notation acting largely as a memory prompt. By the eighteenth century, notation had settled into a European-wide conventional form, though still reliant on a close knowledge of local musical practices for its decoding. That standard form of presenting musical notation has persisted to the present day, despite a huge increase in the diversity of the types of music and musical practices that it has to represent. It is now completely impossible for musicians to have sufficient knowledge of the contexts of all the pieces with which they are confronted to be able to guarantee that they can decipher and perform the notation in real-time.

The development of the study of reading processes has been much slower in the music domain than in the realm of natural languages, and is limited when compared with the exploration of the reading of formal languages (such as mathematical or scientific notation). In his pioneering work in the 1940s Weaver^2^ established that the spacing and harmonic grouping of musical notes were important factors in the guidance of visual fixations (pauses, in his terminology). The work of Sloboda in the 1970s established parallels between language and music readers in terms of structured grouping practices superseding single item identifications in fluent reading^3,4^. Subsequently, studies of eye-voice (or eye-action) spans in reading found similarities in the limitations imposed by the temporal gap between visual processing and motor output for activities such as reading aloud (a gap of a word or two), typing (about six characters) and music sight-reading (about two beats in simple melodies)^5,6^. These studies also established resemblances in *perceptual* span for music and language reading tasks. This span encompasses the whole region around the fixation point from which useful (reading) information can be extracted, and is modulated by the underlying structure of the material.

For these performance-based and time-constrained tasks, the integration of information from a structurally significant region of vision seems to be the strategy followed by skilled readers. Studies of expertise (a recurrent theme in the music-reading literature) have confirmed that skilled sight-reading is associated with an ability rapidly to perceive structured groupings in the score, with experienced musicians performing comparisons of visually presented sequences with fewer (and shorter) glances between patterns^7,8^. Other findings provided by much of the expertise literature are, however, somewhat limited in their application as they tend to merely confirm that sight-reading skill is related to extensive practice (particularly if practice starts at early age, as proposed by Kopiez & Lee^9,10^). As Zhukov^11^ recently noted, simply advocating regular and extensive practice cannot be considered an efficient or elegant approach to learning, from a pedagogical perspective.

An alternative approach to enhancing sight-reading abilities by changing the presentational format of musical notation has, to these authors’ knowledge, not been systematically attempted. Sloboda, writing in the 1970s, was even then able to note that, in contrast, the amount of research on the effects of the presentation of printed language texts on reading fluency was considerable^12^. For example, Bouma^13^ found that interference with adjacent letters conferred a pre-eminence on initial and final symbols in a word in its correct recognition and processing (since these outer letters had a white non-interfering space on one of their sides). This word contour salience could have arisen, however, because of semantic processing, as Bouma found that numbers of correctly identified letters were higher in words than in random letter-strings (undermining the case for the use of gaps in music, which is conventionally held not to manifest propositional or semantic meaning^14^). But studies of specific neurological impairments have led to models of processing of scripts of formal languages (specifically in respect of numerical processing) that can include non-semantic transcoding pathways^15^. It could be the case, therefore, that the inclusion of white spaces as a chunking device for the facilitation of the recognition and processing of musical symbols could work despite the apparent absence of the semantic dimension.

Neuroscientific evidence points in the same direction; mappings of the Visual Word Form area in the left fusiform gyrus have detected responses to written stimuli at this site independently of their semantic content^16^ —albeit with orthographic sensitivity, that is, with preference for contextually regular (or plausible) combinations of symbols^17,18^. Consistent with these data, Mongelli *et al*.^19^ found that music-induced responses did not differ from those elicited by words in this part of the left fusiform gyrus. This multimodal activation did not hold, though, for “non-grammatical” images (*i.e*., those initially not susceptible of being integrated into hierarchically superordinate units such as pictures of houses or faces), and the implication seems to be that it is this hierarchically integrative, combinatorial, quality of letters or notes in certain strings of events that leads to activation of the area^20,21^.

The visual structuring of information so as to facilitate its identification and processing has been a major preoccupation from the point of view of text designers. Highlighting prosodic features in a text (*e. g*., adjusting line breaks to coincide with pauses; emboldening words to indicate stress) has been shown to enhance comprehension, particularly for children at critical points of their learning processes^22^. Visually structuring a text has also proven strongly to affect the format of recalls (with the recalls almost inevitably replicating the original structure of presentation)^23^ —though not their quality. While the high-level design of a text can affect its comprehension, the format of its recall, or the interest of the reader^24^, it does not seem to have a highly significant effect in terms of reading or scanning speed^25,26^. On the contrary, the role of word boundaries (of white spaces between words) has consistently been proven to be central to reading fluency. Filling spaces between words can disrupt the guidance of the next eye movement (particularly the initial landing positions) and the identification and processing of a fixated word^27,28^.

Of particular relevance for our research is the abundant recent literature showing that white spaces facilitate legibility even in writing systems that do not conventionally implement them, as is the case of several Asian languages. Research on interword spacing in Chinese texts, for instance, suggests that its novel implementation enhances reading performance^29,30^. Furthermore, inserting interword spacing into Chinese texts seems to help readers to learn new words. This has been shown for native speakers^31^ and for second language learners^32^; moreover, the effect correlates positively with prior lack of experience with the language^33^. Inserting structuring spaces could therefore be a highly appropriate strategy for music readers, since very few —other than members of a professional elite— could be considered native users of the idioms that they are performing.

A series of preliminary experiments on music reading was undertaken to compare sight-reading of conventional scores and scores implementing modified spacing and separation rules. The results from these preliminary studies indicated that sight-reading scores that included spacings reflecting the underlying structure of the music significantly reduced the numbers of performers’ mistakes and enhanced the fluency of performances^34,35^. The experiment reported here explores the impact of individual design components of the novel scores. In preliminary experiments the novel scores included changes in spacing in at least three levels: phrase separation; sub-phrase separation; and note separations. In light of recent literature on multimodal neuromappings of the Visual Word Form area, and on the beneficial use of interword spacing on language fluency, and of the preliminary results, it was hypothesized that the most effective visual cue in the modified scores should be the inclusion of white gaps at a sub-phrase level. Boundaries at this level should integrate short, manageable and idiomatic groupings of notes, possibly equivalent to the types of informational units marked as words in language.

The experiment was thus divided into three exercises, each including one different spacing modification in the novel scores. In each exercise, each performer (performers being students of percussion from two leading professional Conservatoires) played two different short pieces of two-part music of equivalent style and difficulty at sight on marimba, one in conventional format and one with altered spacing. Each piece was performed twice consecutively in the same version, giving a first and second reading. The order of presentation of the versions (conventional or modified) and of the pieces (piece one or piece two) was counterbalanced across performers. In the first exercise, musical phrasing determined what was included in each line of the altered musical score (*i.e*., scores included only one phrase per line —see Exercise 1); in the second exercise, small white spaces positioned vertically across the staves separated sub-phrasal units in the novel version (see Exercise 2); and in the third, the spacing between notes in the altered version reflected their durational value, following what is known as ‘proportional spacing’ (see Exercise 3).

## Results

Performances were coded for mistakes in the Pitch domain (wrong pitches, failed or weak attacks, omitted notes, added notes) and in the Rhythm domain (wrong durations, hesitations, omitted notes, added notes). These were then added to form Total numbers of mistakes, which were compared for two attempts (Reading 1, Reading 2) for each version (Conventional, Modified).

Generalized Linear Models were implemented with Version (Conventional or Modified), Reading (1st or 2nd reading) and Order of Presentation (Conventional then Modified, or *vice versa*) as independent variables.

### Exercise 1

**Figure 1:**
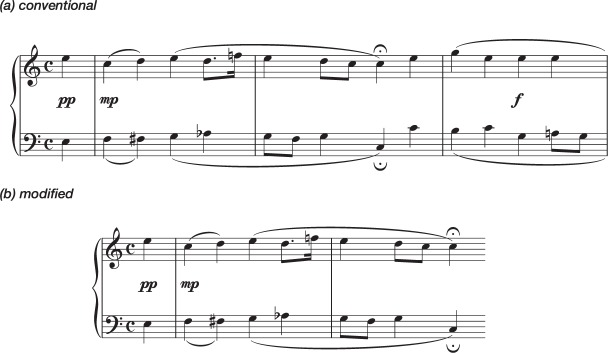
EXERCISE 1. First System of Piece 1 —an arrangement of J. S. Bach’s Chorale nr. 025 in the Richter collection: (**a**) Conventional Version, using the layout and spacing of the Richter 1976 Edition for Breitkopf; (**b**) Modified Version, separating phrasal units (it should be noted that the modified version included only one phrase per line; the first system, shown here, included therefore only the notes up to the first *fermata*). Both versions were presented to participants in A3 paper size in portrait orientation, with staff size of 14 mm. For full examples see Supplementary Information.

Performances with Conventional versions were compared with performances with Modified versions, in which larger structural units (musical phrases) were fitted into single lines of notation (see Fig. 1). It should be noted that in music the term ‘phrase’ is used to indicate an expressive unit that can vary greatly in length, whereas in linguistic terminology it indicates a short group of words standing together as a conceptual unit. For the specific repertoire used in this experiment, most musicologists would use the term to indicate the complete set of notes between two *fermatas* (verse pauses, marked with a  sign) finishing in a cadence, and it is in that sense that it is used here (though it might be argued that the musical phrases here would be comparable, in completeness and length, to a level of structure denoted by the linguistic term ‘sentence’).

#### Main Effects

The Model showed a highly significant effect of Version [χ^2^(1, *n* = 21) = 15.15, *p* < 0.001], a highly significant effect of Reading [χ^2^(1, *n* = 21) = 48.95, *p* < 0.001], and no significance for the Order of Presentation [χ^2^(1, *n* = 21) = 0.02, *p* = 0.865]. The significance for Version reflected better performances with the Conventional Version.

#### Interactions

**Figure 2:**
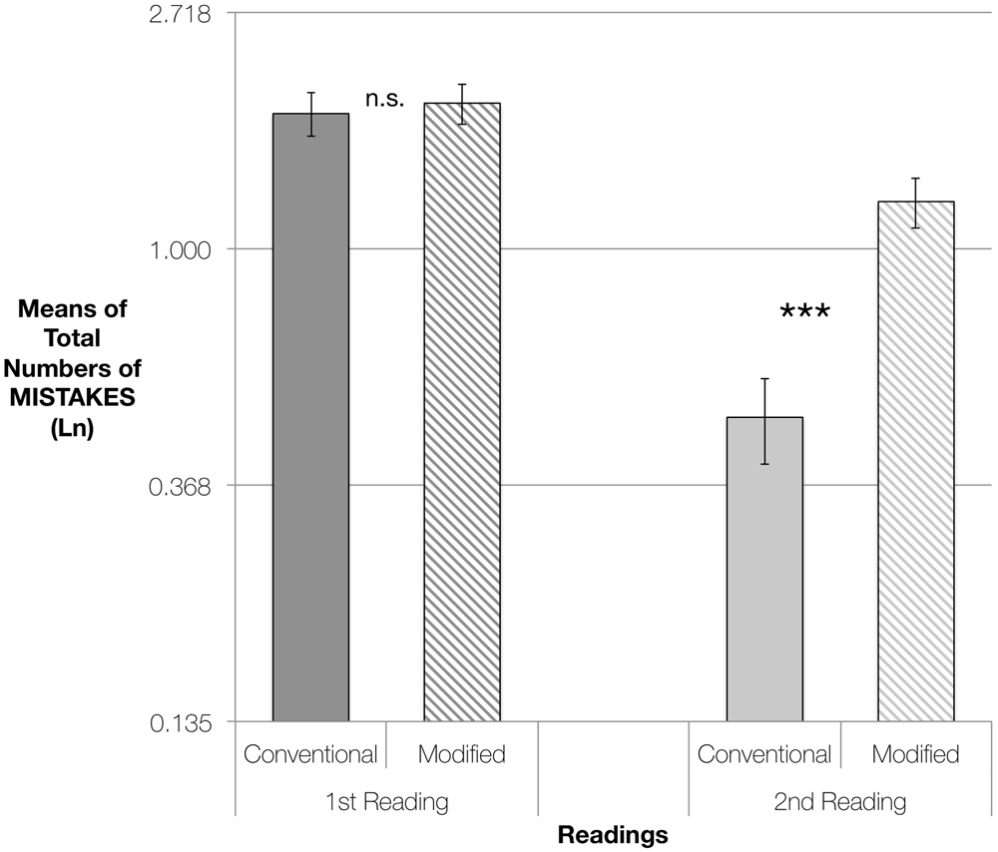
EXERCISE 1: Pairwise Comparisons of Estimated Marginal Means of Total Numbers of Mistakes, for performances using Conventional Versions or Modified Versions of the scores. From left to right, comparisons of mistakes made in the 1st readings and in the 2nd readings. The vertical axis represents logarithmically transformed values (Ln), having used the Ln of the difficulty of each piece as an offset variable. Error bars represent Standard Error. Significance using the Sidak adjustment for multiple comparisons: n. s. = non significant; ***p < 0.001.

The effect of Version by Reading was highly significant [χ^2^(1, *n* = 21) = 12.54, *p* < 0.001], with the 2nd reading showing clear differences between the versions [*p* < 0.001] whereas the first readings showed no significant difference [*p* = 0.733] (see Fig. 2).

#### Order Effect

The interaction between Version, Reading, and Order of Presentation was analysed: the comparisons of estimated marginal means showed a trend towards the versions performing better when presented later in the test (both the Conventional and the Modified Versions eliciting slightly fewer mistakes when the other version had been read first, and this trend being somewhat more marked in the second reading); however, no clear significance was attained in any specific pairwise comparison [all *p*s > 0.050].

### Exercise 2

**Figure 3:**
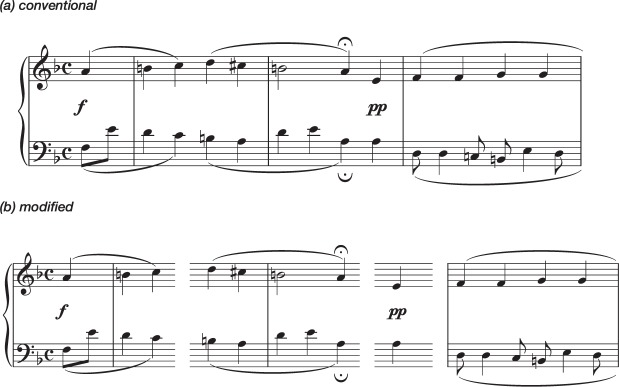
EXERCISE 2. First System of Piece 1 —an arrangement of J. S. Bach’s Chorale nr. 021 in the Richter collection: (**a**) Conventional Version, using the layout and spacing of the Richter 1976 Edition for Breitkopf; (**b**) Modified Version, separating sub-phrasal units. Both versions were presented to participants in A3 paper size in portrait orientation, with staff size of 14 mm.

Performances with Conventional versions were compared with performances with Modified versions, in which short structural units (at sub-phrase level in musical terms: perhaps analogous to word separations in language scripts) were separated by vertical white spaces (see Fig. 3).

#### Main Effects

The Model showed a highly significant effect of Version [χ^2^(1, *n* = 21) = 37.33, *p* < 0.001], a highly significant effect of Reading [χ^2^(1, *n* = 21) = 89.24, *p* < 0.001], and a significant effect for the Order of Presentation [χ^2^(1, *n* = 21) = 8.97, *p* = 0.003]. As hypothesized, the significance for Version reflected better performances with the Modified Versions.

#### Interactions

**Figure 4:**
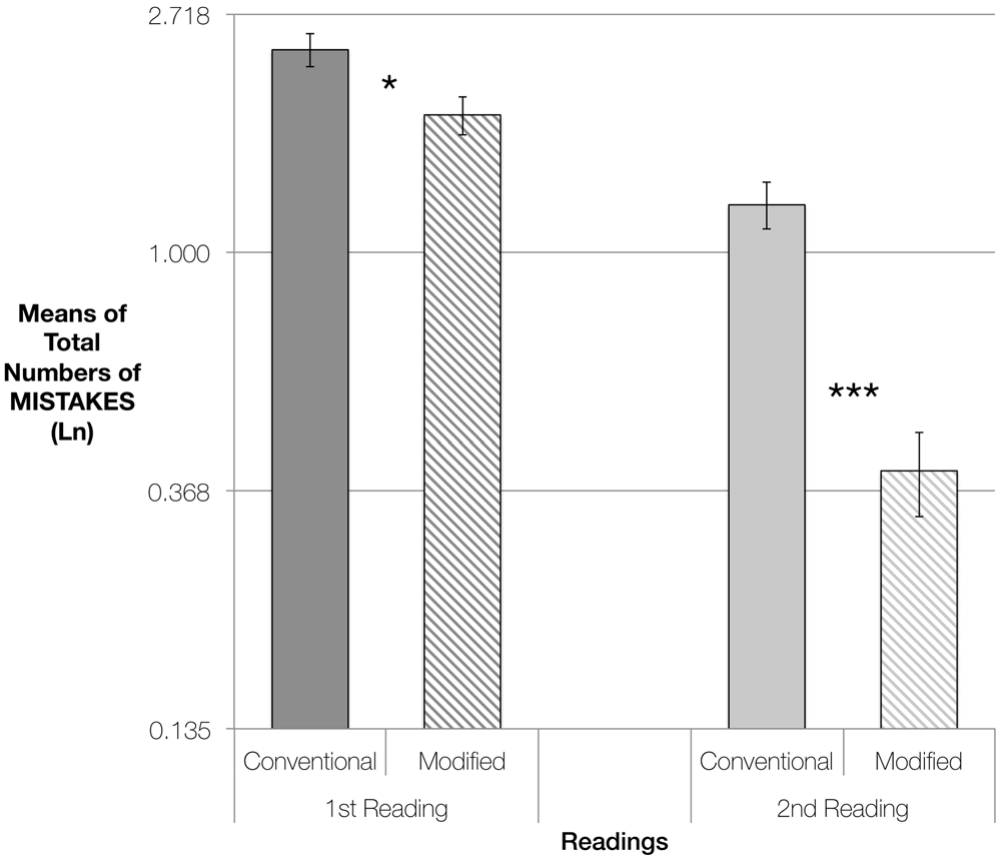
EXERCISE 2: Pairwise Comparisons of Estimated Marginal Means of Total Numbers of Mistakes, for performances using Conventional Versions or Modified Versions of the scores. From left to right, comparisons of mistakes made in the 1st readings and in the 2nd readings. The vertical axis represents logarithmically transformed values (Ln), having used the Ln of the difficulty of each piece as an offset variable. Error bars represent Standard Error. Significance using the Sidak adjustment for multiple comparisons: *p < 0.050; ***p < 0.001.

The effect of Version by Reading was highly significant [χ^2^(1, *n* = 21) = 13.65, *p* < 0.001], with the 2nd reading showing highly significant differences between the versions [*p* < 0.001 whereas the first readings showed significant differences, but not to the same degree [*p* = 0.010] (see Fig. 4).

#### Order Effect

The interaction between Version, Reading, and Order of Presentation was analysed: the comparisons of estimated marginal means showed no particular trend, with differences being non-significant [all *p*s > 0.050].

### Exercise 3

**Figure 5:**
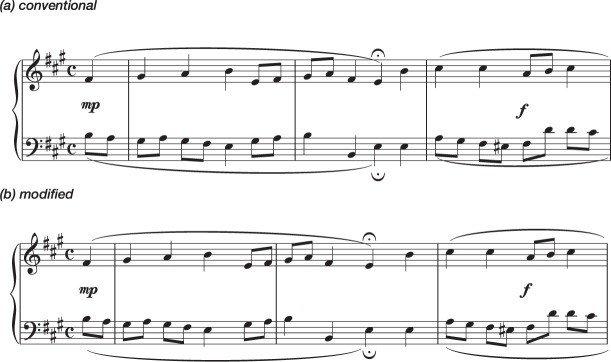
EXERCISE 3. First System of Piece 1 —an arrangement of J. S. Bach’s Chorale nr. 008 in the Richter collection: (**a**) Conventional Version, using the layout and spacing of the Richter 1976 Edition; for Breitkopf (**b**) Modified Version, separating symbols (using ‘proportional notation’). Both versions were presented to participants in A3 paper size in portrait orientation, with staff size of 14 mm.

Performances with Conventional versions were compared with performances with Modified versions, in which notes were separated by spaces proportional to their duration (‘proportional notation’) (see Fig. 5).

#### Main Effects

The model showed no significant effects either for Version [χ^2^(1, *n* = 21) = 0.01, *p* = 0.930] or Order of Presentation [χ^2^(1, *n* = 21) = 0.82, *p* = 0.364], but a significant effect of Reading [χ^2^(1, *n* = 21) = 54.58, *p* < 0.001].

#### Interactions

**Figure 6:**
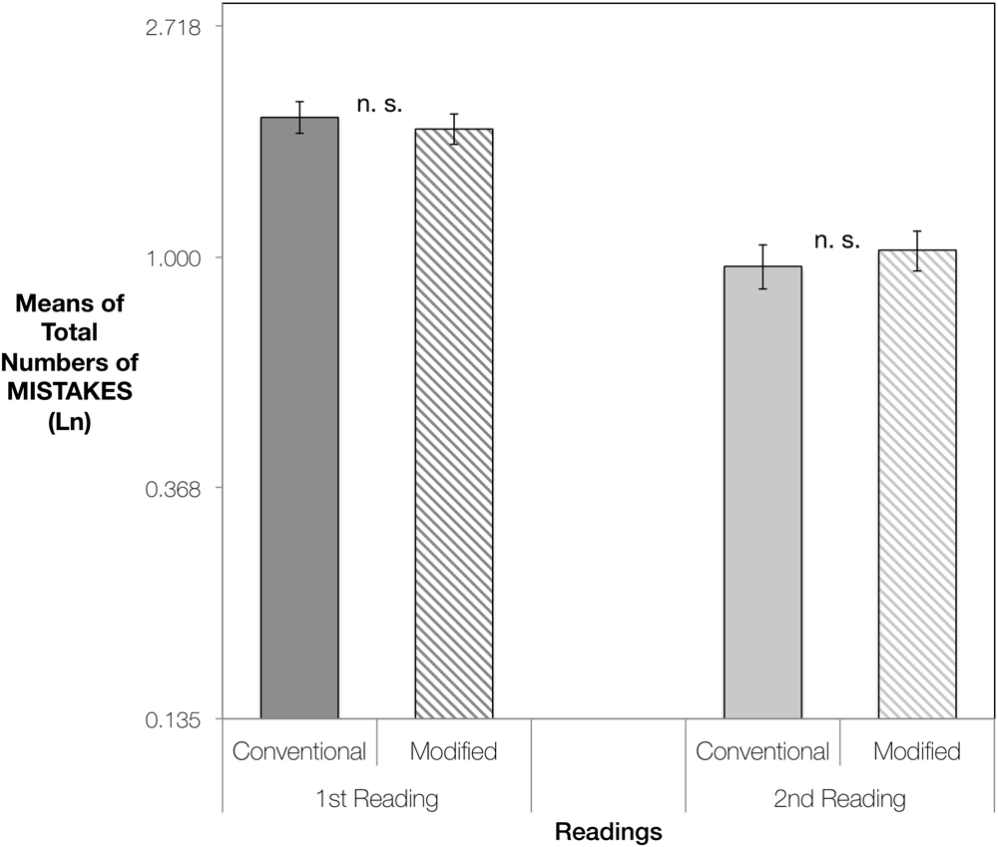
EXERCISE 3: Pairwise Comparisons of Estimated Marginal Means of Total Numbers of Mistakes, for performances using Conventional Versions or Modified Versions of the scores. From left to right, comparisons of mistakes made in the 1st readings and in the 2nd readings. The vertical axis represents logarithmically transformed values (Ln), having used the Ln of the difficulty of each piece as an offset variable. Error bars represent Standard Error. Significance using the Sidak adjustment for multiple comparisons: n. s. = non-significant.

The effect of Version by Reading was non-significant [χ^2^(1, *n* = 21) = 0.54, *p* = 0.459], with first and second readings showing equally non-significant differences (see Fig. 6).

## Discussion

Musical scores were performed with significantly fewer mistakes when white spaces were introduced across the staves to separate sub-phrasal informational units ranging from one to eight beats (with groupings of 3 and 5 beats being the most frequent), in line with estimates of the perceptual span.

Studies on language reading have consistently shown a remarkable impact of interword separation on reading fluency. For instance, the filling of interword spaces with letters has proven to produce considerable interference in the reading process^27^, specifically with word identification and eye movement control^28^. Conversely, the facilitatory effect of including interword spacings has been found even for languages conventionally not implementing it, such as Chinese^29,30^, being particularly effective for Second Language and inexperienced readers^33^.

The importance of interword spacing has also been considered in historical analysis, and some authors (prominently Saenger^36–38^) see, in the transition from continuous unseparated scripts (the *scriptura continua* used in Ancient Greek and Latin) to the use of spaces between words from the viii^th^ century on, a determining aspect of the configuration of Modern European culture. Saenger considers this process so dramatically influential on the relation between reader and text that he proposes that “[…] it is unsurpassed by any other alteration in the act of reading between the patristic age and the xvi^th^ century”. According to Saenger’s view, transformations in calligraphy, dissemination, or even printing techniques would all be subsidiary to the crucial introduction of spacing, which would be inextricably linked to the modern conception of literacy.

Neurophysiological studies of the Visual Word Form Area in the occipitotemporal cortex similarly afford an image of word contours as determinant in the visual encoding of information from a text (*cf* the findings of Brodsky & Kessler^39^ that music reading is facilitated by notationally-intrinsic cues as to contour). The area has been found to be robustly and invariantly activated in the presence of written text, independently of factors like letter case^17^, text position in the visual field^40^, the use of printed or hand-written scripts^41^, and even variations in reading direction or type of character^42^. This robust and selective tuning to integrated groupings of symbols in the area is, however, dependent on literacy, and has been pointed out as a remarkable example of the brain reorganizing to accommodate a novel cultural skill. Dehaene *et al*.^43^ have proposed that literacy acquisition improves early visual processing and reorganizes ventral occipitotemporal pathways: responses to written characters are increased in the left occipito-temporal sulcus, whereas responses to faces shift towards the Right Fusiform Area.

Taken together, the results from behavioural paradigms, socio-historical interpretations, and neurophysiological mappings all seem to ascribe a decisive role in the acquisition of literacy and reading fluency to the ability to perceive integrated and controllable groupings of symbols above and beyond their individual sequential identification. By introducing separations in musical scores at a grouping level that is within the perceptual span of music readers, at a level comparable to word separations, we could be facilitating this ability in the musical domain.

A major and obvious objection to our approach could be the apparent lack of semanticity (at least, in the form of consensual referentiality) in music, in comparison to language. However, turning to the neurophysiological evidence, the attuning of the VWFA to text has shown sensitivity to orthographic regularity, with activations increasing for stimuli forming *approximations* to real words^44^. Moreover, studies on general reading ability have shown skilled readers to be characterised by an ability to access memory codes for *any* (contextually) meaningful visual patterns, for instance non-semantic line drawings^45,46^. An unequivocal, language-like, semanticity does not therefore seem to be an excluding criterion for the activation of these neural pathways.

It was notable that other spacing novelties introduced in different exercises did not enhance the fluent performance of these musical scores; separating phrases by lines (including only one phrase per line) had in fact a detrimental effect. This result seems to agree with those in the literature on linguistic texts showing that design and layout can affect focus (*e. g*., in terms of student engagement^47^) and selective information retrieval (for instance in examination-type situations^48^), but do not directly affect reading fluency^25,26^. In the general presentation of a text, familiarity with the format used will, however, play a major role^23,24^. It has been demonstrated, for instance, that *a priori* expectations greatly increase the benefits subjects gain from preview in parafoveal vision^49^. In the particular case of the pieces presented here (arrangements of Bach Chorales), even if they did not form part of the core repertoires studied by these musicians (as would have been the case with organists, or singers, for example), benefits that might have been offered by the introduction of novel design layouts did not outweigh the advantages afforded by the familiarity of the conventional layout.

Furthermore, as Rayner & Pollatsek^5^ have noted, the gap between visual processing and motor output in sight reading is not very great, and is much more restricted than what conventional wisdom in music might suggest (this gap is moreover similarly small in activities as reading aloud and typing). Organising the musical text visually to encompass chunkings of whole phrases (of eight beats, in the particular pieces at hand) might be working against the cognitive constraints of the participants. Rayner & Pollatsek^5^ have suggested that a major limitation on tasks that require translation of complex inputs into a continuous motor transcription could be short-term memory. If the encoding process gets too far ahead of the output, as was possibly the case in Exercise 1, there is likely to be a deterioration in the representation of the material stored in the buffer. It might be, though, that with professional performers (*i. e*., not students, as in the present case) to whom the idiomatic conventions of a certain style are thoroughly familiar, the predictability of melodic lines and their harmonic implications would lead to encoding that would encompass whole phrases. Future research should look at the relation between expertise and ideal length of integration/separation, as the perceptual span has been linked to expectations^49^, familiarity with materials^50^, general reading skills^5^, or more specific abilities such as auditory representation capacities^8^.

Again, it was notable that separating single notes to reflect their implicit duration (“proportional notation”) had no observable effect. It could be suggested that, in the case of the proportional notation, the difference between the conventional and modified versions of such rhythmically simple music was too small to have an effect. Moreover, we had used the default note spacing rule of *Sibelius 7.1.3* (the music processing software employed to produce the scores) to specify spacing at a reference note value level (here, eighth notes). Durations of other notes were thus spaced proportionately as multiples or submultiples of eighth notes. *Sibelius’s* rules represent industry standards; it would be rare to encounter proportionate notation scaling occupying significantly more space than was used here. It may be that this reflects an issue in text design where there seems to be an interplay between letter separation and word separation, such that an increase in letter separation beyond a standard value can lead to a decrease in word identification performance. Whether a threshold for, or a balance between, proportional note separation and identification of groupable units exists in music is an issue that requires to be investigated in the future.

Overall, these results point to strategies for enhancing the readability of musical scores at a professional level and in pedagogical contexts. Professionally, composers may be able to package their ideas in ways that meet with more immediate comprehension —and hence, more fluent and accurate presentation— by performers. Performers may be able to use technology (in the form of music-processing software and digital music stands) to customise the scores they have to read so as to enhance readability under the pressures of concert performance or recording. In the modern world of music performance, time is money, and the time allocated to rehearsals has decreased significantly; these modifications should help ease the financial pressures on performance by making the process of rehearsal substantially more efficient. Pedagogically, these results suggest ways of facilitating the acquisition of musical literacy amongst children and beginning readers, particularly in the light of results showing that the reading speed of second-language learners (though not that of native readers) is increased by the insertion of inter-word spaces^33^; making reading music easier should help those motivated to develop musical expertise to a high level, and it could enhance the intrinsic motivations of those for whom music reading is a painful chore. These results also contribute to a growing body of literature that supports the notion that the physical separation of meaning units facilitates comprehension in texts or symbolic systems in general, irrespective of their domain of application.

## Methods

### Ethical approval

All participants were students at the Spanish Conservatoires where the experiments took place. Neither of the institutions where the experiments were undertaken operate a research ethics protocol themselves, nor do they have formal links to local institutions where ethical review could appropriately have been conducted. Accordingly, it was agreed with the Heads of the relevant Department in those institutions that ethical review should be referred to the Research Ethics Committee of the Faculty of Music, University of Cambridge, which has extensive experience in considering ethical implications that may arise from historical, ethnographic, and scientific research involving music; the experiments received approval as being in accordance with the guidelines and regulations framed in the University of Cambridge’s Research Ethics policy. (see https://www.research-integrity.admin.cam.ac.uk/research-ethics).

Participation in the experiment was completely voluntary, was not remunerated, and was organised and supervised by their instrumental teachers (see Acknowledgements). The main experimental procedures were described to the participants in advance so that they were informed about what to expect. Written consent was obtained for participation in the test and for use of the recordings. Participants were informed that they could withdraw from the research at any time. They were also informed of the general purpose of the study (a study of sight-reading abilities in relation to performance materials), but remained naive (even after finishing their participation) as to the research hypothesis —that the modified scores would elicit better performances. When the whole experiment was completed (all participants had been recorded), they were debriefed as to the purpose of the study.

### Participants

Twenty-one Percussion students (two female) took part in individual sessions of approximately 60 minutes, in which they read musical pieces at an ability-dependent tempo. These students (aged between 18 to 24) were recruited from all degree years (from Probationary to Masters) at the *Escuela Superior de Música de Aragón*, in Saragossa, and at the *Escuela Superior de Música de Cataluña*, in Barcelona. All participants understood and followed the protocols, and no data had to be discarded. All participants reported normal or corrected-to-normal vision, and none reported cognitive (reading) difficulties. Percussionists were chosen because we aimed to control, as far as possible, for possible effects of familiarity with performance repertoire and musical idiom on reading accuracy and fluency. The pieces used (arrangements of Bach’s chorales) are in a style that is core to common Western musical practice and hence is aurally and conceptually familiar to all, but, at the same time, is not part of the usual performance repertoire for percussionists.

### Experimental materials

#### Pieces

The pieces to be read were two-voice transcriptions for Marimba of Bach chorales from the Richter collection^51^: nrs 025 and 266 in Exercise 1; nrs 021 and 317 in Exercise 2; nrs 008 and 012 in Exercise 3. The Soprano part was kept in the original octave, in the right hand stave; the Bass was transcribed on its own in the left hand stave with changes of octave when needed to not to encompass a too large range in the instrument.

Two pieces of very similar level of difficulty were used in each Exercise (one presented as a conventional score; the other using modifications). An experimental design with two similar scores rather than one repeating readings in different versions of the same piece was favoured so as to avoid the unwanted ‘mistake permanence’ effect detected in previous experiments. This distortion whereby a particular note, cell or passage that is problematic in a first reading will tend to elicit problems in subsequent readings is something that is common in any musician’s experience, but that was not taken into account in the design of our initial experiments: we followed an intuitive or direct paradigm design of having a performer read the *same* piece of music in different versions, but this meant that the possible effect that a given version would have on the number of mistakes would be strongly modulated by how correct the *previous* performance had been. This effect has furthermore been thoroughly observed in the language reading literature^52,53^.

The pieces were slightly tweaked, within idiom, so as to make them contain an equal number of notes per Exercise (an equal number of onsets: 53 onsets for pieces 1 and 2 in Exercise 1; 52 onsets in Exercise 2; 61 In Exercise 3; see Supplementary Information). Ideally, the difficulty of both pieces should be as similar as possible, so that ‘Piece’ could then be factored out when comparing the mean levels of performance for the two versions. In statistical analysis it is of course possible to implement a model using Piece as an Offset Variable, but obtaining results without an effect of Piece is more desirable, for two reasons: 1) the quantification of the impact of the piece is always going to be approximate (even using a logarithmic scaling as is usually done in this cases); and 2) the presentation of the results will not have such a clear and directly apprehensible quality as with absolute measures.

#### Presentation

Both the conventional and the modified versions were presented in portrait orientation, in A3 paper size (usual for percussionists, as the music stand is normally separated from the player by a large instrument), with the staves printed starting from the upper margins at 4 cm in both cases. All scores used staves of exactly the same size (14 mm, measured as the vertical distance between the first and the fifth line). The size of notes and all other musical symbols were also exactly the same in all scores. Materials were created using Sibelius 7.1.3 on a MacBook Pro running OSX 10.10. The Music Fonts used were all from the ‘Opus Standard’ family —the default used by the Sibelius programme— and the same for all scores (see examples in Figs 1, 3 and 5; see all scores in Supplementary Information).

Participants were accompanied by a metronome throughout the first phrase; the metronome was switched off at the first *fermata* (pause sign in the score). Participants were requested to try to complete the rest of the exercise at the same tempo, as is the case in any sight-reading exercise implemented, for instance, in conservatoire examinations or orchestral auditions.

#### Dynamic markings

These were added to the original texts. The dynamics were implemented following an analysis of the grammar (harmonic progressions, cadences and modulations) of each piece, with a traditional emphasis on deviations from the tonic and (suggested) modulations. This would further serve as a test of additional information retrieval, on top of pitch and rhythm encoding.

Furthermore, the dynamic markings substituted the text (‘lyrics’) that normally is included in chorales (but would be irrelevant in this instrumental test), and that influences in conjunction with many other factors the design decisions of conventional editions. The number of markings is therefore somewhat higher than what would be usual in this kind of repertoire but, at any rate, exactly the same for both versions.

#### Phrasing marks

The Conventional versions included phrasing slurs that indicated exactly the same chunking or parsing of the materials as suggested with the separation of basic discourse units by space in the modified versions. These slurs are not commonly included in conventional editions of this repertoire (although the symbology is completely standard in other scores) but were introduced here so as to not to afford an encoding advantage to the modified version that could otherwise be easily expressed with regular musical symbols.

#### Instrument

Good quality Marimbas (standard size, not large/concert size) were provided by both conservatoires - instruments that all participants were familiar with. The choice of mallets was left to the participants, both in terms of hardness (although medium-hard was recommended) and number (two or four), since what was of interest was the difference in performance between versions for a given performer, not the particular achievements or style of that performer.

### Procedure

**Figure 7:**
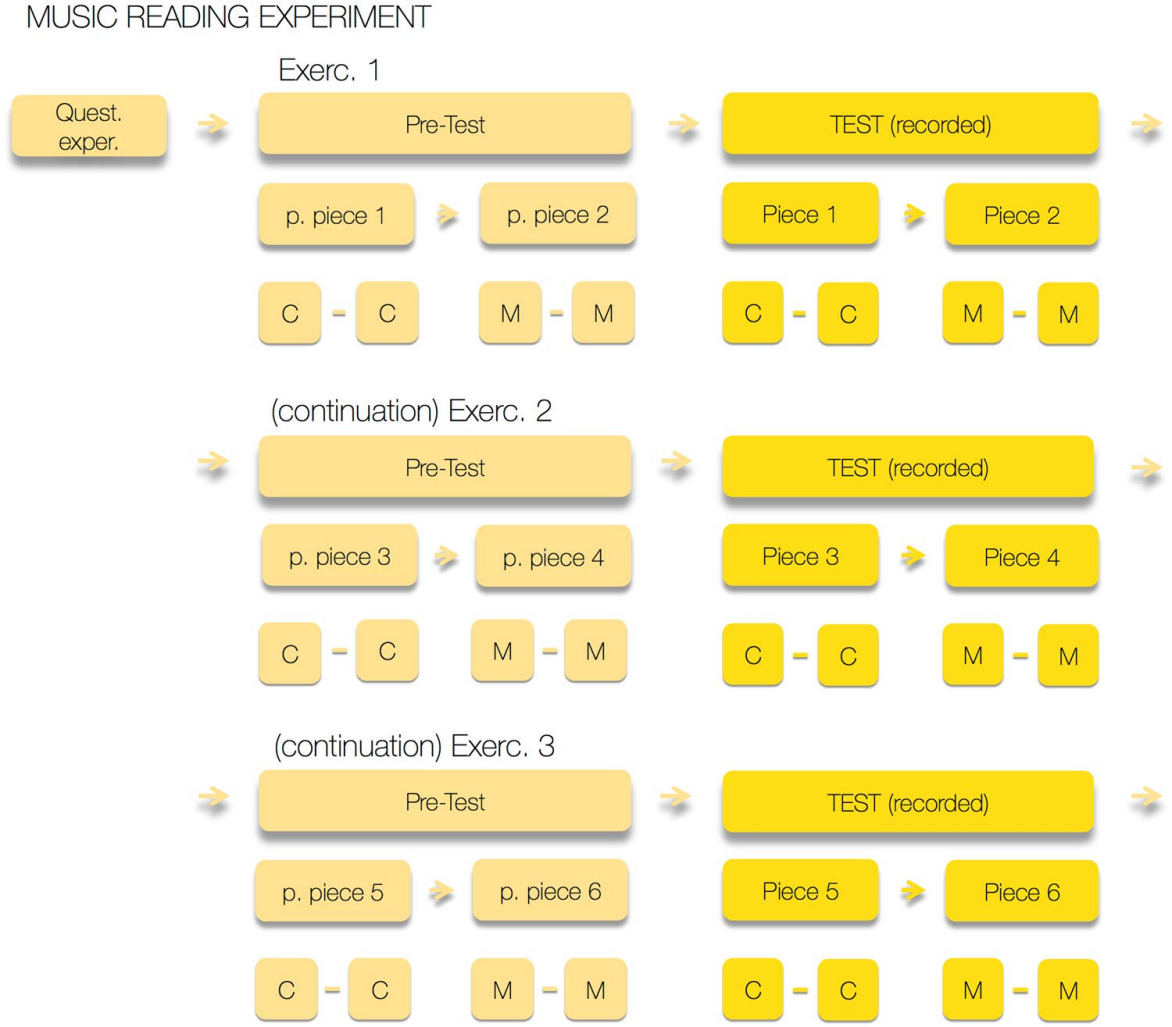
Schematic timeline for Participant 1: the participant is questioned on musical experience and reading ability predictors, and then completes three reading exercises. Each exercise consists of a Pre-test and the recorded Test. In the pre-tests participants are presented with practice pieces of similar difficulty and appearance to the pieces to be performed in that exercise. The participant played thus 12 short pieces of music, with six of the performances being coded. The order of presentation of Exercises (Exerc. 1, Exerc. 2, Exerc. 3) pairs of pieces within them (*e. g*., in Exercise 1, Piece 1/Piece 2) and of Versions (Conventional/Modified) was counterbalanced throughout the rest of participants. Approximate duration: 60 min.

Individual sessions with the participants lasted approximately 60 minutes, with almost 20 minutes dedicated to each Exercise. Figure 7 shows a schematic timeline for the full experimental procedure.

Each individual session started with a questionnaire to help establish a general level of reading competence of the participant.

A pre-test (within each exercise, the initial 10 minutes) was then used with a view to familiarize the participant with the procedure and materials, and to judge the subject’s sight-reading abilities more in detail, since this would determine an individual tempo at which he/she would perform the test.

The pre-test included two complete readings of two preparatory pieces, allowing for the trial of four different tempos. The aim was to find a tempo for each participant that would be at the required threshold of his/her abilities. This had to be pondered carefully, particularly since each piece was to be read two times in the subsequent test: too slow a tempo would prompt a ceiling effect, specially in the second reading, which is not ideal for analysis (the differences, if any, would not be statistically significant); and too fast a tempo can block the participant completely, not allowing him/her to complete the task, or can lead to performances with such a high number of errors that data show violations of normality that can not easily be corrected/transformed.

The pre-test also served to introduce participants to the modified scores. The design of the modified scores was very briefly explained with the following protocol:

“In these scores we have introduced a series of graphical modifications departing from conventional layout and spacing rules, and we are wondering if this could possibly enhance the readability of the music. These new engraving rules follow the underlying structures of the piece, marking its phrases, motifs, and note-spacings. Thus, you will see that each system includes only one phrase/the subdivisions of a phrase are marked with white gaps/the score uses proportional notation (whilst pointing at the relevant elements of the score in each case)”.

After the pre-test, before starting the recorded test, each participant was instructed with the following protocol:

“You will be presented with a series of musical pieces that you should read at the best of your abilities. In the pre-test I looked for a tempo at which I think you will be out of your comfort zone, so do not worry if you make mistakes - for research purposes these mistakes are useful. But at any rate, do not stop and go back to where you made a mistake. Imagine that this is a live situation. As in a live situation, your aim is also to try to keep the initial tempo throughout.”

No scrutiny period was allowed for between the unveiling of the score (by taking away a covering sheet) and the start of the metronome. At the start of the metronome, participants counted three beats and commenced playing, with a late start being considered as a mistake. The metronome was left on for the duration of the first phrase (until the first *fermata*); participants were instructed to keep the same tempo until the end of the piece, stopping to rest only at the *fermatas* at the end of each phrase. This procedure had also been rehearsed in the pre-test.

### Coding

#### Numbers of mistakes

All the audio clips of the participants’ performances were marked for mistakes in the Pitch and the Rhythm domain, which were then added up, to produce a Total number of mistakes for each performance. In both the Pitch and Rhythm domains, one error was counted for each deviated, wrong, eliminated or added item.

In the Pitch Domain, a Deviation could be a hit on the edge of the key, producing a noticeably bad quality of sound, or a hit in the gap between the intended key and the adjacent one, whereas a Wrong item would be produced by playing an altogether different pitch to the one written.

In the Rhythm Domain, a Deviation would be marked when there was a noticeable tempo alteration but the proportions between note values were not changed, whereas a Wrong marking would be used when an altogether different value was played.

The major, most noticeable errors, produced by eliminating a note altogether or adding notes that were not written, counted thus as two in the Total of errors, since they were disruptive both in the Pitch and in the Rhythm Domain.

Participants were accompanied by a metronome throughout the first phrase; the metronome was switched off at the first *fermata* (pause sign in the score). Participants were requested to try to complete the rest of the exercise at the same tempo, as is the case in any sight-reading exercise implemented, for instance, in conservatoire examinations or orchestral auditions.

### Statistical analysis

Analyses implementing Generalized Linear Models, assuming a Log (*i. e*., non-linear) Link Function, were run for the Total Numbers of mistakes. The probability assumed was Poisson, as the dependent variable consisted of counts of events expressed in a limited range of positive integers.

Based on comments by several participants (stating that certain keys were more difficult to play for them than others), the means of mistakes per Piece were firstly explored, and notable differences were found in spite of our precautions when designing the materials. Apparently - although not in relation to any specific instrumental idiosyncrasies of mallet percussion (as was confirmed by the teachers) - this set of participants found the Piece in a-minor easier to perform than the piece in C-Major (in Exercise 1), the piece in e-minor easier than the piece in d-minor (in Exercise 2), and the piece in A-Major easier than the piece in Eb-Major (in Exercise 3) (see Supplementary Information, for full examples.). A recurring comment at the sessions was that ‘music with flats is more difficult’ but, as stated, this is not generalizable, nor can it be related to any limitation or specificity of mallet instruments or their repertoire.

At any rate, this meant that Piece had to be integrated in the analysis as an Offset Variable (with its coefficient not estimated by the model). Since each participant would have different levels of exposure to piece difficulty, the Ln of the difficulty of each piece (accounted in Means of mistakes) was used as to balance other effects^54^. Reliable main effects and interaction effects could thus be calculated. However, for the pairwise or multiple comparisons in the interactions analyses, Estimated Marginal Means were thus expressed in logarithmic values, and therefore rankings and significance values of differences rather than specific means are reported here (as logarithmic values of means do not immediately help to visualize comparisons).

## Supplementary information

Supplementary information: Musical scores
