# Supplementary material for: White spaces, music notation and the facilitation of sight-reading
Source: Sci Rep. 2019 Mar 28;9:5299. doi: 10.1038/s41598-019-41445-1 (PMC6439220; doi:10.1038/s41598-019-41445-1)
Supplement: Supplementary file 1 — Supplementary information: Musical scores [file 41598_2019_41445_MOESM1_ESM.pdf]

Supplementary information: Musical scores

Title of the manuscript. White spaces, music notation and the facilitation of sight-reading

Author list.

1. Arild Stenberg
2. Ian Cross

Academic affiliation. For both authors: Centre for Music and Science, Faculty of Music, University of Cambridge, UK.

Exercise 1

Piece 1

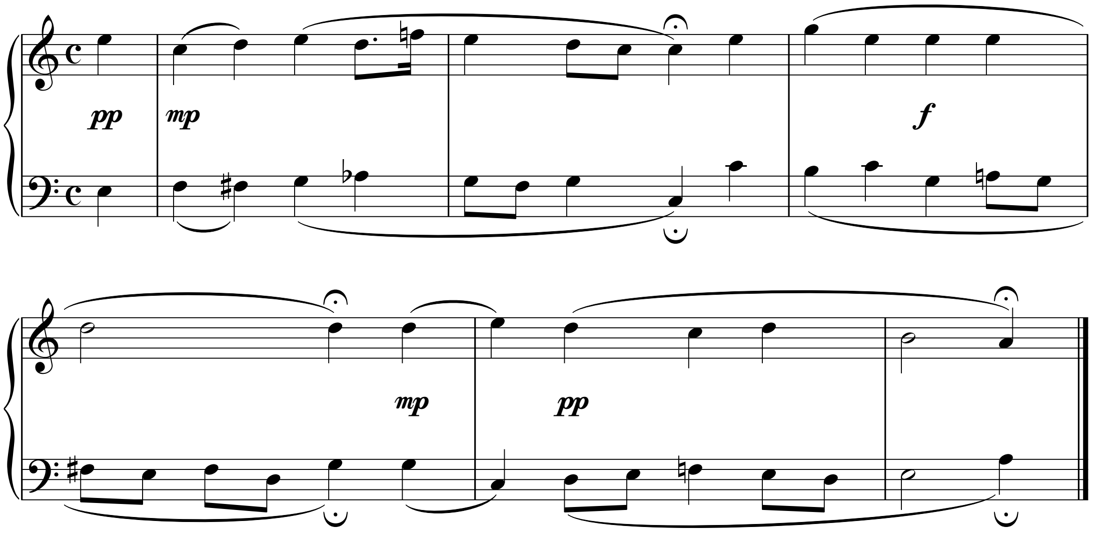

Figure 1.1.a.

EXERCISE 1 - PIECE 1: J. S. Bach's Chorale, nr. 025 in the Richter collection; CONVENTIONAL Version, using the layout and spacing of the Breitkopf 1976 Edition.

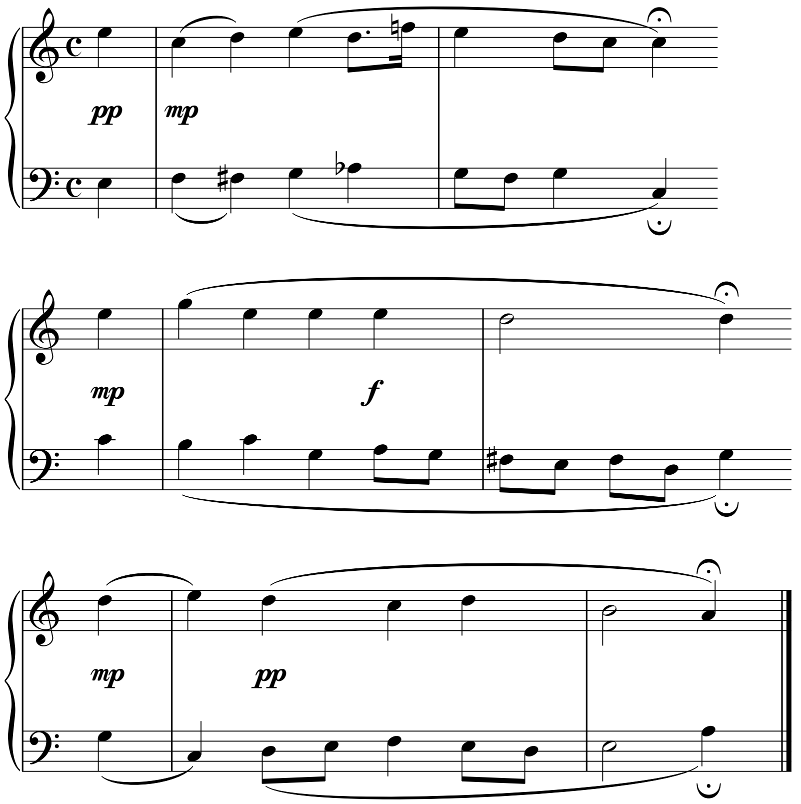

Figure 1.1.b.

EXERCISE 1 - PIECE 1: J. S. Bach's Chorale, nr. 025 in the Richter collection; MODIFIED Version, separating only *phrasal* units.

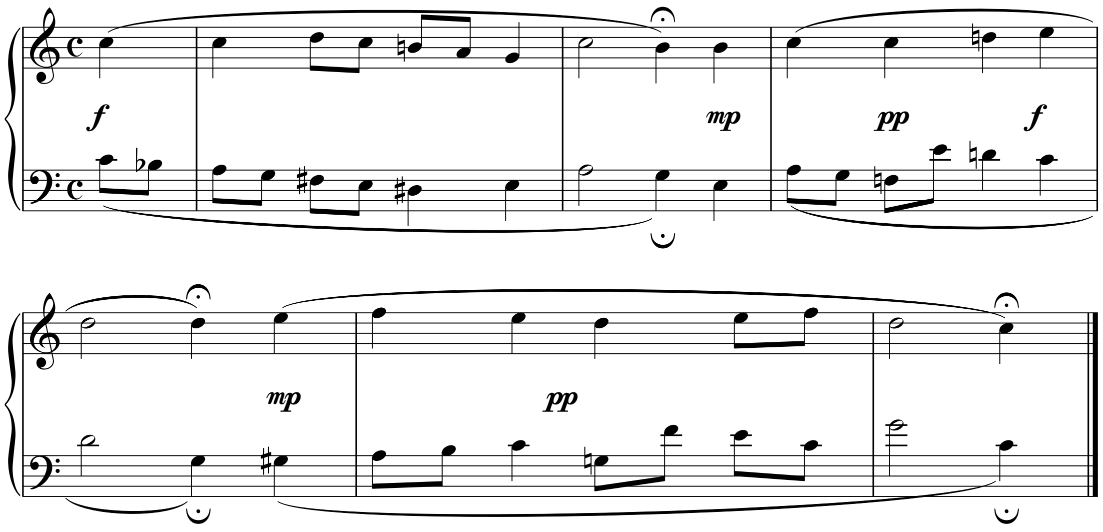

Figure 1.2.a.

EXERCISE 1 - PIECE 2: J. S. Bach's Chorale, nr. 266 in the Richter collection; CONVENTIONAL Version, using the layout and spacing of the Breitkopf 1976 Edition.

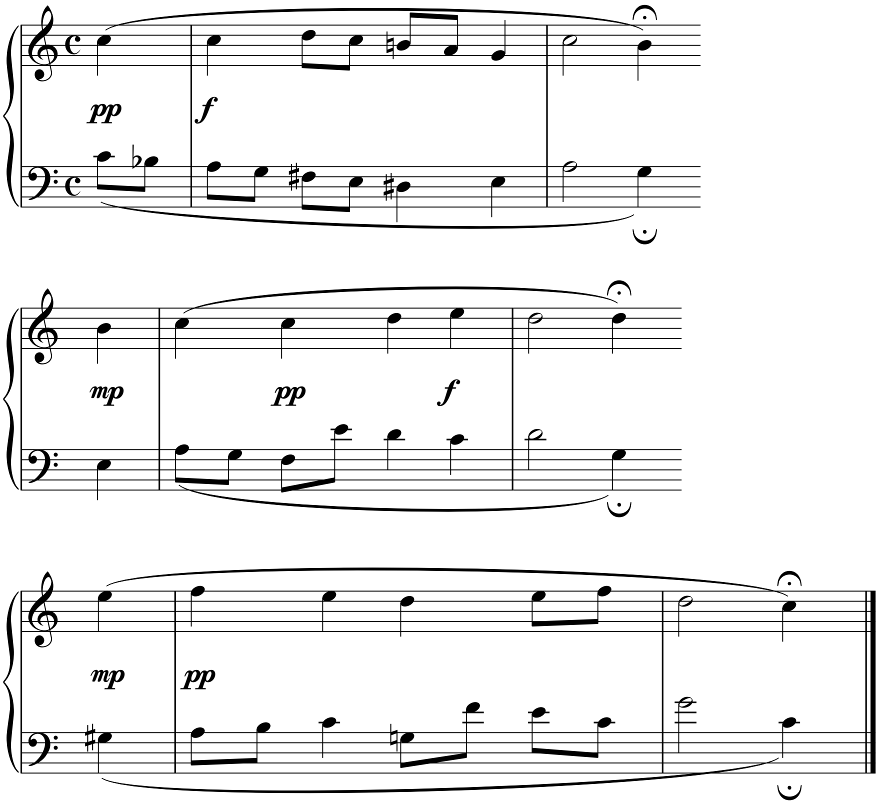

Figure 1.2.b.

EXERCISE 1 - PIECE 2: J. S. Bach's Chorale, nr. 266 in the Richter collection; MODIFIED Version, separating only *phrasal* units.

Exercise 2

Piece 1

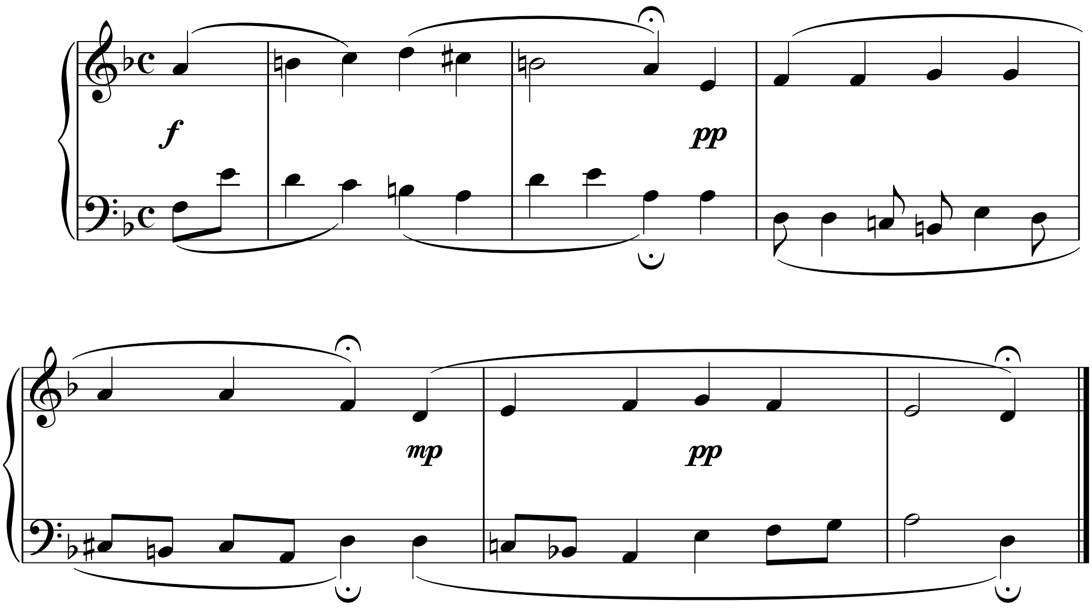

Figure 2.1.a.

EXERCISE 2 - PIECE 1: J. S. Bach's Chorale, nr. 021 in the Richter collection; CONVENTIONAL Version, using the layout and spacing of the Breitkopf 1976 Edition.

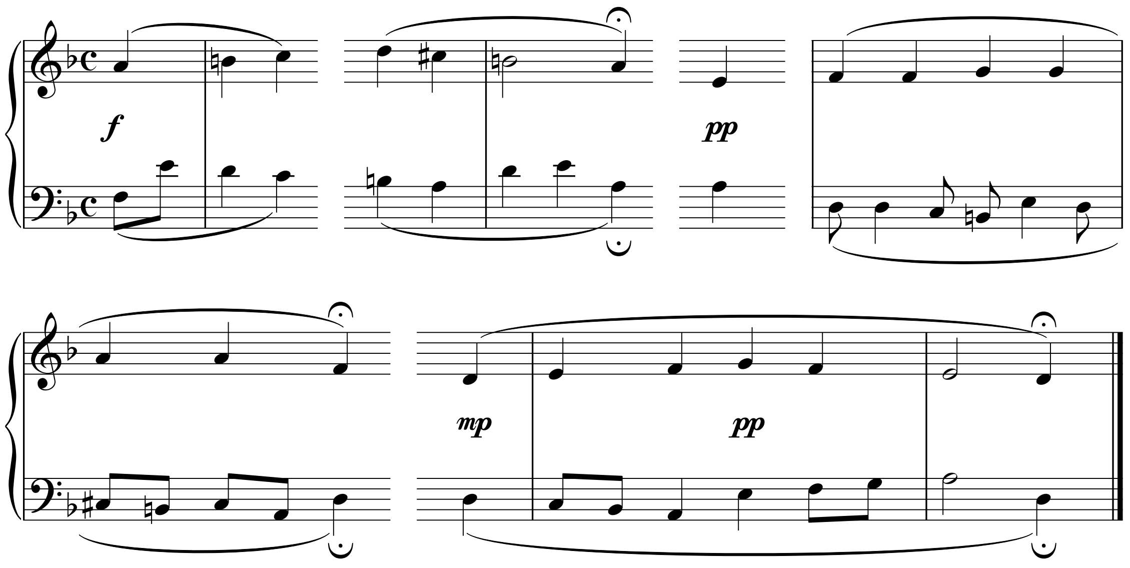

Figure 2.1.b.

EXERCISE 2 - PIECE 1: J. S. Bach's Chorale, nr. 021 in the Richter collection; MODIFIED Version, separating only *sub-phrasal* units.

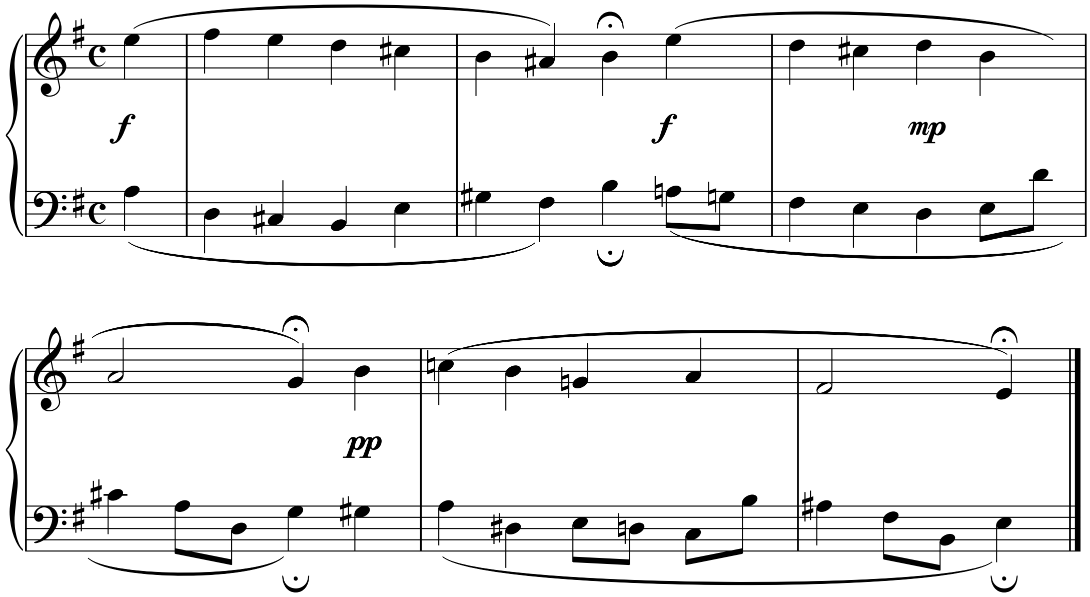

Figure 2.2.a.

EXERCISE 2 - PIECE 2: J. S. Bach's Chorale, nr. 317 in the Richter collection; CONVENTIONAL Version, using the layout and spacing of the Breitkopf 1976 Edition.

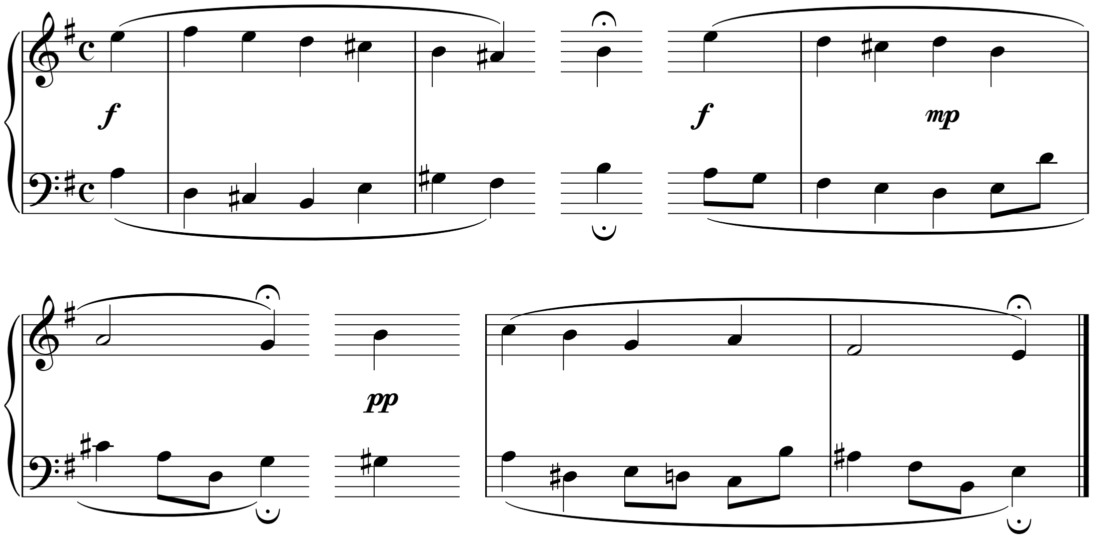

Figure 2.2.b.

EXERCISE 2 - PIECE 2: J. S. Bach's Chorale, nr. 317 in the Richter collection; MODIFIED Version, separating only *sub-phrasal* units.

Exercise 3

Piece 1

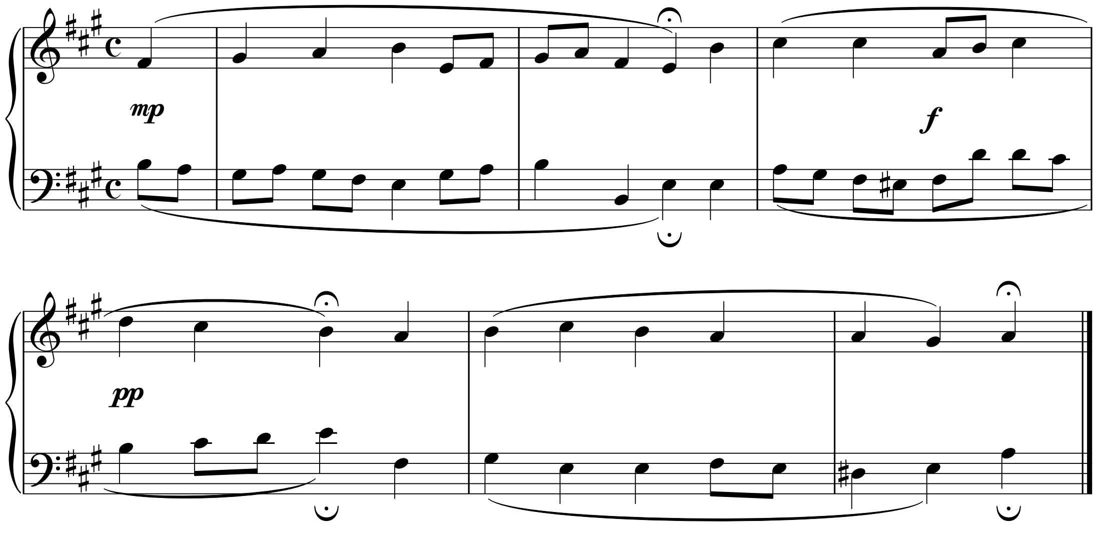

Figure 3.1.a.

EXERCISE 3 - PIECE 1: J. S. Bach's Chorale, nr. 008 in the Richter collection; CONVENTIONAL Version, using the layout and spacing of the Breitkopf 1976 Edition.

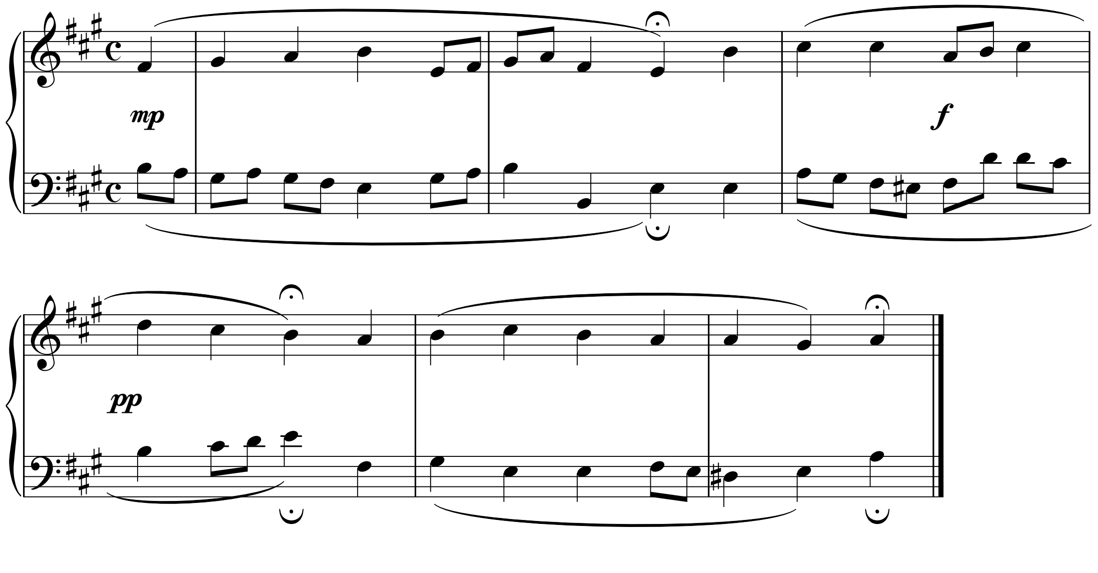

Figure 3.1.b.

EXERCISE 3 - PIECE 1: J. S. Bach's Chorale, nr. 008 in the Richter collection; MODIFIED Version, separating only *symbols* ('proportional notation').

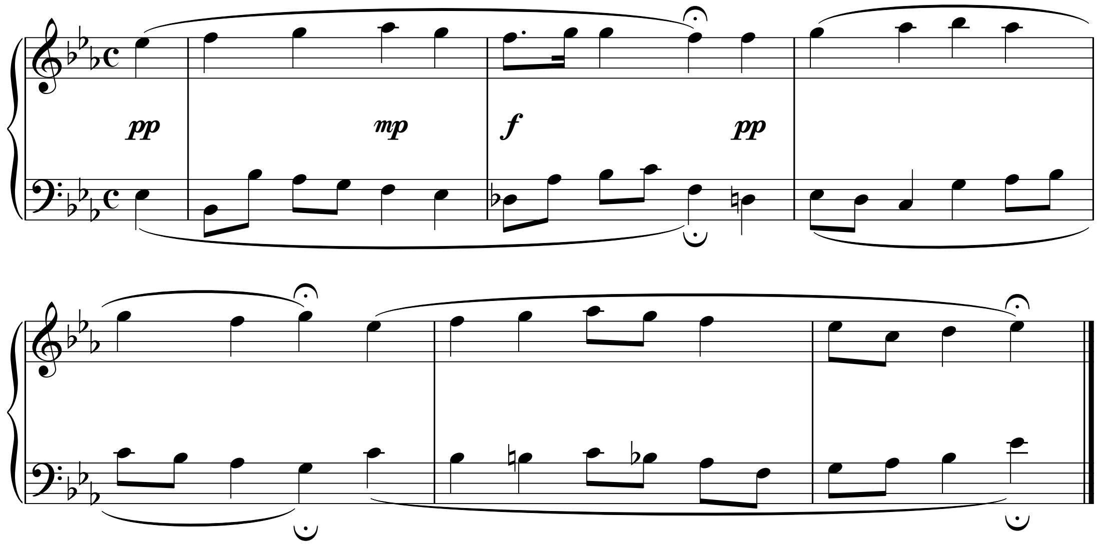

Figure 3.2.a.

EXERCISE 3 - PIECE 2: J. S. Bach's Chorale, nr. 012 in the Richter collection; CONVENTIONAL Version, using the layout and spacing of the Breitkopf 1976 Edition.

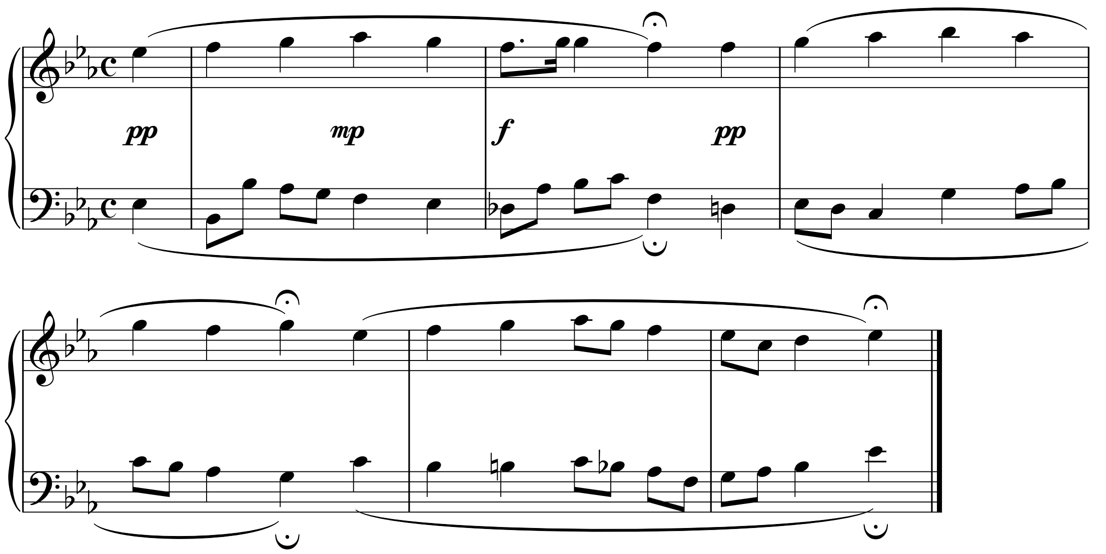

Figure 3.2.b.

EXERCISE 3 - PIECE 2: J. S. Bach's Chorale, nr. 012 in the Richter collection; MODIFIED Version, separating only *symbols* ('proportional notation').
